# Supplementary material for: Comparable Effectiveness of Cefuroxime and Piperacillin-Tazobactam as Empirical Therapy for Methicillin-Susceptible Staphylococcus aureus Bacteremia
Source: Microbiol Spectr. 2022 Apr 19;10(3):e01530-21. doi: 10.1128/spectrum.01530-21 (PMC9241907; doi:10.1128/spectrum.01530-21)
Supplement: SUPPLEMENTAL FILE 1 — Supplemental material. Download spectrum.01530-21-s001.pdf, PDF file, 0.1 MB [file spectrum.01530-21-s001.pdf]

## Supplementary material

**Supplementary Table 1:** Overview of different models of adjustment and their association with 30-day mortality using Akaike Information Criterion

| | K | AICc |
|---|---|---|
| Baseline model | 17 | 2744 |
| Adjustment for all baseline model variables except smoking | 16 | 2763 |
| Adjustment for all variables except alcohol and smoking | 15 | 2883 |
| Adjustment for all variables except injection drug abuse and smoking | 14 | 3565 |
| Adjustment for all variables except alcohol, injection drug abuse and smoking | 13 | 4999 |
| Adjustment for all variables except alcohol, injection drug abuse, smoking and year of administration | 12 | 4997 |
| Adjustment for all variables except alcohol, injection drug abuse, smoking and the indication of therapy | 7 | 5043 |

K: number of parameters in the model; AICc: calculated Akaike Information Criterion score. Smaller AICc equals better fitting model; baseline model: adjustment of age, sex, CCI score, the indication of therapy, year of administration, alcohol abuse, i.v. drug abuse and smoking

**Supplementary Table 2:** Empirical antimicrobial therapy administered other than cefuroxime or piperacillin/tazobactam in the combination therapy group

| | Total (n = 392) | Oral therapy (n = 43) |
|---|---|---|
| Ciprofloxacin (%) | 145 (39.5) | 7 (16.3) |
| Gentamycin (%) | 116 (29.6) | - |
| Vancomycin (%) | 71 (18.1) | - |
| Meropenem/ertapenem (%) | 63 (16.1) | - |
| Cloxacillin/dicloxacillin/flucloxacillin (%) | 35 (8.9) | 4 (9.3) |
| Penicillin/ampicillin/amoxicillin (%) | 32 (8.2) | 6 (14.0) |
| Clarithromycin/roxithromycin (%) | 14 (3.6) | 9 (20.9) |
| Rifampicin (%) | 9 (2.3) | 7 (16.3) |
| Fusidic acid (%) | 5 (1.3) | 4 (9.3) |

**Supplementary Table 3**: Crude and adjusted hazard ratios of 7-, 30- or 90-day mortality or 90-day relapse for patients with methicillin-susceptible *S. aureus* bacteremia receiving empirical therapy with cefuroxime monotherapy, piperacillin/tazobactam monotherapy or combination therapy with either cefuroxime or piperacillin/tazobactam

| | 7-day mortality | | 30-day mortality | | 90-day mortality | | 90-day relapse | |
|---|---|---|---|---|---|---|---|---|
| | Crude HR (95 % CI) | Adjusted HR (95 % CI) | Crude HR (95 % CI) | Adjusted HR (95 % CI) | Crude HR (95 % CI) | Adjusted HR (95 % CI) | Crude HR (95 % CI) | Adjusted HR (95 % CI) |
| Cefuroxime monotherapy (n = 337) | 1.00 | 1.00 | 1.00 | 1.00 | 1.00 | 1.00 | 1.00 | 1.00 |
| Piperacillin/ tazobactam monotherapy (n = 429) | 1.19 (0.83-1.73) | 1.03 (0.60-1.75) | 1.22 (0.93-1.60) | 1.09 (0.71-1.66) | 1.33 (1.06-1.68) | 0.98 (0.71-1.37) | 2.08 (0.92-4.71) | 1.51 (0.52-4.33) |
| Cefuroxime comb. therapy (n = 195) | 0.83 (0.50-1.37) | 0.96 (0.52-1.74) | 0.92 (0.65-1.32) | 1.13 (0.69-1.84) | 0.91 (0.66-1.23) | 1.10 (0.75-1.62) | 2.17 (0.86-5.50) | 1.97 (0.62-6.32) |
| Piperacillin/ tazobactam comb. therapy (n = 197) | 1.16 (0.74-1.81) | 1.10 (0.59-2.06) | 1.28 (0.93-1.77) | 1.08 (0.67-1.75) | 1.23 (0.92-1.64) | 1.14 (0.77-1.69) | 1.73 (0.65-4.61) | 1.48 (0.42-5.18) |

**Supplementary Table 4**: Characteristics of the propensity score-matched methicillin-susceptible *Staphylococcus aureus* bacteremia cases stratified by empirical monotherapy with cefuroxime or piperacillin/tazobactam

| | Cefuroxime monotherapy (n = 237) | Piperacillin/tazobactam monotherapy (n = 214) | P-value |
|---|---|---|---|
| Female (%) | 93 (39.2) | 82 (38.3) | 0.92 |
| Median age (IQR) | 74 (62-84) | 74 (61-84) | 0.81 |
| Comorbidity score (CCI) | | | |
| *Low, CCI = 0* (%) | 52 (21.9) | 32 (15.0) | |
| *Medium, CCI = 1-2* (%) | 88 (37.1) | 81 (37.9) | |
| *High, CCI > 2* (%) | 97 (40.9) | 101 (47.2) | 0.14 |
| Median SOFA score at onset (IQR) | 2 (1-4) | 3 (1-4 | 0.92 |
| Pitt score ≥ 4 at onset (%) | 18 (7.6) | 11 (5.1) | 0.38 |
| Indication of EAT | | | |
| *Fever with unknown focus* (%) | 58 (24.5) | 66 (30.8) | |
| *Skin, soft tissue or bone infection* (%) | 45 (19.0) | 25 (11.7) | |
| *Urinary tract infection* (%) | 24 (10.1) | 27 (12.6) | |
| *Iv device infection* (%) | 7 (3.0) | 2 (0.9) | |
| *Pneumonia* (%) | 61 (25.7) | 56 (26.2) | |
| *Not mentioned* (%) | 23 (9.7) | 28 (13.1) | |
| *Other* (%) | 19 (8.0) | 10 (4.7) | 0.073 |

**Supplementary Table 5**: Characteristics of methicillin-susceptible *Staphylococcus aureus* bacteremia cases stratified by empirical monotherapy with cefuroxime or piperacillin/tazobactam administered (A) less than three days (B) or three days or more

(A)

| | Cefuroxime monotherapy < three days (n = 150) | Piperacillin/ tazobactam monotherapy < three days (n = 304) | P-value |
|---|---|---|---|
| Female (%) | 60 (40.0) | 119 (39.1) | 0.94 |
| Median age (IQR) | 73.5 (59-82) | 75 (62-84) | 0.35 |
| Comorbidity score (CCI) | | | |
| *Low, CCI = 0* (%) | 36 (24.0) | 47 (15.5) | |
| *Medium, CCI = 1-2* (%) | 50 (33.3) | 124 (40.8) | |
| *High, CCI > 2* (%) | 64 (42.7) | 133 (43.8) | 0.064 |
| Smoking (%) | 33 (22.0) | 66 (21.7) | 0.99 |
| Daily alcohol consumption (%) | 28 (18.7) | 75 (24.5) | 0.23 |
| Injection drug use (%) | 7 (4.7) | 11 (3.6) | 0.97 |
| Any immunosuppression* (%) | 7 (4.7) | 18 (5.9) | 0.69 |
| Median SOFA score at onset (IQR) | 3 (1-4) | 3 (1-4) | 0.72 |
| Pitt score ≥ 4 at onset (%) | 13 (10.3) | 14 (5.4) | 0.12 |
| Secondary manifestations | | | |
| *Endocarditis* (%) | 7 (4.7) | 35 (11.5) | 0.028 |
| *Osteomyelitis* (%) | 10 (6.7) | 12 (3.9) | 0.30 |
| *Spondylodiscitis* (%) | 11 (7.3) | 18 (5.9) | 0.71 |
| *Arthritis* (%) | 9 (6.0) | 14 (4.6) | 0.68 |
| *Meningitis* (%) | 0 (0.0) | 2 (0.7) | 0.81 |
| *Pneumonia* (%) | 11 (7.3) | 20 (6.6) | 0.92 |
| *Other* (%) | 9 (6.0) | 10 (3.3) | 0.27 |
| Relapse within 90 days (%) | 4 (2.7) | 11 (3.6) | 0.80 |
| 7-day mortality (%) | 38 (25.3) | 54 (17.8) | 0.078 |
| 30-day mortality (%) | 54 (36.0) | 95 (31.2) | 0.36 |
| 90-day mortality (%) | 63 (42.0) | 134 (44.1) | 0.75 |

IQR: interquartile range

*HIV positive, chemotherapy, other immunosuppressive treatment or other immunosuppression

(B)

| | Cefuroxime monotherapy ≥ three days (n = 187) | Piperacillin/ tazobactam monotherapy ≥ three days (n = 125) | P-value |
|---|---|---|---|
| Female (%) | 80 (42.8) | 56 (44.8) | 0.81 |
| Median age (IQR) | 73 (62-85) | 72 (60-84) | 0.59 |
| Comorbidity score (CCI) | | | |
| *Low, CCI = 0* (%) | 43 (23.0) | 13 (10.4) | |
| *Medium, CCI = 1-2* (%) | 73 (39.0) | 49 (39.2) | |
| *High, CCI > 2* (%) | 71 (38.0) | 63 (50.4) | 0.0095 |
| Smoking (%) | 46 (24.6) | 41 (32.8) | 0.22 |
| Daily alcohol consumption (%) | 31 (16.6) | 33 (26.4) | 0.067 |
| Injection drug use (%) | 2 (1.1) | 4 (3.2) | 0.23 |
| Any immunosuppression* (%) | 13 (7.0) | 3 (2.4) | 0.38 |
| Median SOFA score at onset (IQR) | 2 (1-4) | 3 (2-4) | 0.28 |
| Pitt score ≥ 4 at onset (%) | 5 (3.6) | 8 (8.0) | 0.23 |
| Secondary manifestations | | | |
| *Endocarditis* (%) | 9 (4.8) | 6 (4.8) | 1.00 |
| *Osteomyelitis* (%) | 5 (2.7) | 2 (1.6) | 0.81 |
| *Spondylodiscitis* (%) | 8 (4.3) | 7 (5.6) | 0.79 |
| *Arthritis* (%) | 7 (3.7) | 2 (1.6) | 0.45 |
| *Meningitis* (%) | 0 (0.0) | 0 (0.0) | NA |
| *Pneumonia* (%) | 14 (7.5) | 10 (8.0) | 1.00 |
| *Other* (%) | 19 (10.2) | 7 (5.6) | 0.22 |
| Relapse within 90 days (%) | 4 (2.1) | 10 (8.0) | 0.030 |
| 7-day mortality (%) | 9 (4.8) | 17 (13.6) | 0.011 |
| 30-day mortality (%) | 32 (17.1) | 35 (28.0) | 0.031 |
| 90-day mortality (%) | 52 (27.8) | 52 (41.6) | 0.016 |
